# Evaluating Quality of Life and Marital Contentment among Seroconcordant and Serodiscordant HIV-Infected Couples in Comparison to Non- HIV Couples

**DOI:** 10.30476/ijcbnm.2021.87420.1430

**Published:** 2021-07

**Authors:** Azam Faraji, Niloofar Namazi, Leila Doryanizadeh, Hadi Raeisi Shahraki

**Affiliations:** 1 Department of Obstetrics and Gynecology, School of Medicine, Shiraz University of Medical Sciences, Shiraz, Iran; 2 Maternal-Fetal Medicine Research Center, Shiraz University of Medical Sciences, Shiraz, Iran; 3 Infertility Research Center, Shiraz University of Medical Sciences, Shiraz, Iran; 4 Department of Midwifery, School of Nursing and Midwifery, Estahban branch, Islamic Azad University, Estahban, Iran; 5 Department of Epidemiology and Biostatistics, School of Health, Shahrekord University of Medical Sciences, Shahrekord, Iran

**Keywords:** HIV, Marital therapy, Quality of life, Sex

## Abstract

**Background:**

Quality of life (QOL) and marital contentment, especially marital satisfaction, are important aspects of life. These items are more important in couples involved in HIV due to the present social stigma among this population considering women more vulnerable. The aim of this study was to determine the QOL and marital contentment status among seroconcordant and serodiscordant HIV couples compared to non-HIV ones.

**Methods:**

In this cross-sectional study, 66 serodiscordant, 74 seroconcordant, and 70 non-HIV couples who referred to Lavan High-risk Behavior Counseling Center, Shiraz during September 2017 and December 2019 were studied. QOL and marital contentment were assessed by World Health Organization Quality of Life-BRIEF (WHOQOL BREF) and ENRICH questionnaire, respectively. Chi-square test for qualitative variables, independent T-test and ANOVA followed by LSD post hoc test for quantitative variables were performed. All statistical analyses were performed using SPSS 19.0, and P<0.05 was set as the significant level.

**Results:**

The score of QOL questionnaire was significantly higher in non-HIV couples than serodiscordant and seroconcordant groups (P<0.001). There was no significant difference among seroconcordant and serodiscordant groups (P=0.99), and infected males vs. females (P=0.13). Non-HIV couples had significantly higher marital contentment in comparison to serodiscordant and seroconcordant groups (P<0.001). No difference was detected among seroconcordant and serodiscordant groups (P=0.81) although more contentment was observed among the males (P=0.01).

**Conclusion:**

Our study revealed that QOL and marital contentment were different among non-HIV and HIV infected couples. Besides, marital contentment was higher among males than female’s in infected patients.

## INTRODUCTION

Human immunodeficiency (HIV) is a global concern, especially among developing countries, with a prevalence of more than 76.1 million infected cases in the world. ^1 , 2^ The prevalence of HIV in Iran is increasing with a range from 18 to 40% in different studies. ^3 , 4^ It is reported that the new diagnosed infected cases are in a decreased curve from 2010 to 2018 with reduction from 2.1 million to 1.7 million new infected cases globally. ^5^ There is a trend to end the HIV epidemic by increased knowledge, accurate programming and promoting access to prevention methods of transmission, and more successful treatment. ^6^

HIV affects people in different areas including sexual, financial, physical and psychological aspects. Quality of life (QOL) can be impressed by the disease in different aspects. Some factors like the patients’ age, educational level and marital status are found to determine its impact. ^7^ Also, marital problem is another disturbing issue among HIV-infected patients. ^8^ Intercourse is known as the major root of transmission accounting for 46.5% of HIV cases; it may become a worrying problem among serodiscordant couples, defined as couples that one is HIV infected and the other spouse is seronegative, rather than seroconcordant couples defined as couples that are both HIV infected. The factor involved in this fact is the fear of altering the serum status in the non-infected individual of serodiscordant couples. ^9^ Considering this fact, the role of information and communication technology is highlighted to improve prevention and behavioral skills. ^10^ Voluntary testing and counseling, especially among high risk patients, are the strategies used to overcome the HIV rate of transmission and stigma. ^11^

HIV-infected women may face with more difficulties to experience good QOL and marital contentment since they are more vulnerable to be affected in the aspects of social stigma, susceptibility to get infected and emotional events. It is unfortunate that some patients refuse to receive treatments due to the shame of being infected. ^12^ These factors may differ in the type of infected couples (seroconcordant, male-infected serodiscordant and female-infected serodiscordant). These aspects, beside the high prevalence of HIV and importance of QOL and marital contentment in this neglected group, prompted us to conduct this study. We aimed to assess QOL and marital contentment, especially marital satisfaction scale, among serodiscordant couples, seroconcordant couples compared to non-HIV ones.

## MATERIALS AND METHODS

This cross-sectional study was performed on three groups of seroconcordant, serodiscordant and non-HIV couples during September 2017 and December 2019. Infected couples (whether one or both infected individuals) were recruited from patients who were referred to Lavan High-risk Behavior Counseling Center, Shiraz, Iran, the only referral center for HIV positive patients, affiliated with Shiraz University of Medical Sciences. The non-HIV couples were recruited from patients who visited gynecologists of Motahari clinic that was affiliated to Shiraz University of Medical Sciences due to routine check-up. Before enrolling, HIV testing of both individuals of each couple was done to categorize them into each group of seroconcordant, serodiscordant and non-HIV. Infection status of patients was tested first by performing rapid test. If it was negative, the patient was labeled as non-HIV. If the test was positive, after rechecking, ELISA test was done to confirm the diagnosis. Inclusion criteria were signing the informed consent, being in a stable married position, living with each other, and not being divorced.

Participants were excluded if they had any chronic disease including liver, kidney, thyroid, neurologic or known psychiatry diseases except for HIV. Also, the patients with malignancy were omitted since in a research it has been shown that some chronic diseases impact the QOL, like diabetes. ^1^

Based on the information of a previous study ^13^ considering the mean difference of 1.3 (d=1.3) and standard deviation of 3.5 and 4.5 for the two groups of seroconcordant (S1) and serodiscordant (S2), while considering type one (α) and two (β) errors of 0.05 and 0.2, the minimum required sample size was estimated as 130 persons in each group (65 couples), according to the following equation. The groups were both individuals positive (seroconcordant), one of the individuals positive (the man or women was positive and the partner is seronegative), and non-HIV couples that both individuals were not infected. ^13^

n=Z1-α2+Z1-β2(S12+S22)d2Considering the possibility of withdrawal from participation, we selected 75 couples in each group. Finally, after the sample drop, 210 couples including 66 serodiscordant, 74 seroconcordant and 70 non-HIV couples were enrolled in the current study. It should be added that in the seroconcordant couples, 22 couples were females with HIV positive living with non-infected male, while in 44 couples, the males were HIV positive and the females were HIV negative.

In this study, three questionnaires were tested and administered by a trained healthcare staff of the mentioned center who was assigned for collecting data. Three questionnaires were used: 1) Demographic data including age, sex, living in urban or rural area, duration of marriage, how the individual got familiar to his/her spouse, root of transmission (risk factor), and number of living children, 2) Assessment of QOL based on the standard certified translated WHOQOL-BREF questionnaire as mentioned, and 3) Assessment of total marital contentment based on standard certified translated Enrich questionnaire as mentioned.

Both individuals of each couple filled out their questionnaire simultaneously to report their last two-week conditions.

WHOQOL-HIV BREF questionnaire has been introduced as a reliable tool to evaluate QOL introduced first by World Health Organization (WHO) at 1998. ^14^ This questionnaire contains a total of 26 questions. 24 items represent 4 main domains including 1) physical domain (7 items) measuring three areas of pain and discomfort, energy and fatigue, and sleep and rest; 2) psychological domain (6 items) measuring five areas of positive feeling; negative feeling, learning and concentration, bodily image, and self-esteem; 3) social domain (3 items) measuring three areas of personal relationships, practical social support, and sexual activity; 4)environmental domain (8 items) measuring five areas of financial resources, health care availability, opportunities for acquiring new information and skills, opportunities for leisure, and transport. The remaining two items describe health related QOL and general QOL (not included in scoring). Scores were calculated based on the questionnaire directory published by WHO organization at 1998. ^14^ The questionnaire was evaluated based on a 5-point Likert scale (Each item scored from 1 to 5 e.g., 1=Not at all to 5=completely). The mean score of the items was calculated for each domain (score 7-35 for physical domain, 6-30 for psychological domain, 3-15 for social domain, and 8-40 for environmental domain). Then, the scores of each domain were converted to a 0-100 scale by the following transformation formula (higher scores denote higher QOL). ^14^

n=xi-minximaxxi-minxi In the version of questionnaire (WHO_QOL-BREF), internal consistency reliability was assessed by Cronbach alpha, which reported 0.82 for physical domain, 0.81 for psychological domain, 0.68 for social domain, and 0.80 for environment domain. To assess discriminant validity, we investigated the ability of domain scores to discriminate between patients from healthy group using t-test to compare mean scores of the two groups. Findings showed that mean scores were significantly different in all the domains (P<0.01). Construct validity was assessed by correlating the domain scores with each general item (R^2^=0.52, standardized β for physical domain=0.323, psychological domain=0.258, social domain=0.102, environment domain=0.171). Comparative Fit Index (CFI) demonstrated acceptable fit for four factor model (CFI reported 0.863 in Random split half sample A, 0.864 in Random split half sample B, 0.876 in sick sample, and 0.868 in well sample). ^15^

We used the Persian translated certified questionnaire that was validated by Usefy et al. in 2010; Cronbach`s alpha for reliability reported 0.81 for physical health domain, 0.78 for psychological domain, 0.82 for social health domain, and 0.80 for environmental domain. The bivariate inter-correlation coefficient of this scale with overall health status (OHS) questionnaire was 0.63 (P<0.001). Also construct validity of the questionnaire was approved using exploratory factor analysis for four factor model. For convergent validity, it was shown that all the questions of WHOQOL were significantly correlated with their original domains in the expected direction (P<0.01). Construct validity was assessed by correlating the domain scores with each general item (standardized β for physical domain=0.517, psychological domain=0.584, social domain=0.442, environment domain =0.502). ^16^

In the current study, the Cronbach`s alpha coefficient for QOL was 0.90, and validity was assessed by correlating the domain scores with each general item (R^2^=0.100, standardized β for physical domain=0.296, psychological domain=0.293, social domain=0.312, environment domain=0.247). 

The marital contentment questionnaire was prepared by David H. Olsonin1989. ^17^ This questionnaire has several forms with different number of questions. In this study, we selected the form of this questionnaire with 35 questions which was translated by Asoodeh et al. in Iran. It has four major domains including idealistic distortion, marital satisfaction, communication and conflict resolution, each having 5, 10, 10 and 10 questions, respectively. This questionnaire was evaluated based on a 5-point Likert scale (1=strongly disagree to 5=strongly agree); higher mean scores show higher marital satisfaction ratings. The scales are answered in five-point Likert scale (from 1 to 5) including strongly disagree, moderately disagree, neither agree nor disagree, moderately agree, and strongly agree. 19 question had reverse scoring (from 5 to 1). The mean score of the items was calculated for each domain (score 5-25 for idealistic distortion, 10-50 for marital satisfaction, 10-50 for communication, and10-50 for conflict resolution). Then, the scores of each domain were converted to percentage by the following transformation formula. A score of 5-100 is assigned for each domain (higher scores denote higher marital satisfaction: 5-15 indicates very low, 20-35 indicates low, 40-60 indicates moderate, 65-80 indicates high, and 85-100 indicates very high. ^18^

n=xi-minximaxxi-minxi Alpha coefficient was reported 0.86for marital satisfaction, 0.80 for communication, 0.84for conflict resolution, and 0.83 for idealistic distortion. The test-retest reliability was reported 0.86for marital satisfaction, 0.81 for communication, 0.90for conflict resolution, and 0.92 for idealistic distortion. ^18^ In the current study, the Cronbach`s alpha coefficient for Marital Satisfaction scale was 0.83and validity was assessed by correlating the domain scores with each general item (R^2^=0.91, standardized β for marital satisfaction=0.297, communication=0.384, conflict resolution =0.334, idealistic distortion=0.022).

Descriptive statistics were reported as mean±SD and number (%) for quantitative and qualitative variables, respectively. To examine the association between qualitative variables, we performed Chi-square or Fisher’s exact test. Also, independent t-test and ANOVA test followed by LSD post hoc test were used to compare the groups in terms of quantitative variables. All the statistical analyses were performed in SPSS 19.0, and P<0.05 was set as significant. The normality assumption was assessed using Shapiro-Wilk test and approved for all the variables under study.

After approval of the study by the Medical Ethics Committee of Shiraz University of Medical Sciences (registration no.IR.SUMS.REC.1396.101) and free counseling, all participants signed the written informed consent. Contribution was voluntary and participants were informed to be permitted to refuse the project without any alteration of their treatment. Privacy of individuals was considered, and the results and the name of the participants were undisclosed in all steps of the project. Individuals were informed of the result.

## RESULTS

The most prevalent educational status in seroconcordant couples was elementary and middle school. High school and diploma degree were the most relevant educational status in the serodiscordant couples whereas Bachelor degree was seen more among non-HIV couples. Couples in the non-HIV group had significantly higher levels of education (P<0.001) and were significantly younger (P=0.007) than the seroconcordant and serodiscordant groups, but there was no significant difference among the serodiscordant and seroconcordant couples. (P=0.007). As to the type of marriage, family selection with individual’s companionship was the most common situation in the seroconcordant, serodiscordant groups and non-HIV couples. But there was no significant difference among the three groups in terms of marriage duration (P=0.08) and number of children (P=0.21). More information about demographic characteristics is summarized in Table 1 .

**Table1:** Comparison of demographic characteristics among the study groups

Variable	Serodiscordant (N=132) N (%)	Seroconcordant (N=148) N (%)	non-HIV (N=140) N (%)	P value
Education				<0.001*
No Education	5 (3.80)	5 (3.40)	0 (0)	
Elementary and Middle School	53 (40.20)	81 (54.70)	6 (4.30)	
High school and Diploma	56 (42.40)	50 (33.80)	46 (32.90)	
Bachelor’ Degree	16 (12.10)	12 (8.10)	70 (50.00)	
Master`s Degree and higher	2 (1.50)	0 (0)	18 (12.90)	
Marriage type				0.02*
Optional	26 (19.70)	43 (29.10)	54 (38.60)	
Family Selection with Individual`s Companionship	88 (66.70)	90 (60.80)	71 (50.70)	
Family Selection with Individual` Opposition	18 (13.60)	13 (8.80)	14 (10.00)	
Obligatory	0 (0)	2 (1.40)	1 (0.70)	
Birthplace				0.006*
Rural	61 (46.20)	57 (38.50)	80 (57.10)	
Urban	71 (53.80)	91 (61.50)	60 (42.90)	
Risk factor			---	<0.001*
Sexual transmission	20 (15.20)	86 (58.10)		
Blood transfusion	34 (25.80)	43 (29.10)		
Others	12 (9.10)	19 (12.80)		
	**Mean±SD**	**Mean±SD**	**Mean±SD**	
Age	39.43±8.36	39.97±8.69	36.66±10.83	0.007**
Marriage duration	8.63±7.62	11.16±9.13	10.10±11.10	0.08**
Number of children	1.27±1.29	1.57±1.54	1.41±1.30	0.21**
Quality of life	73.03±15.23	73.01±14.11	85.72±7.15	<0.001**
Marital Contentment	68.19±18.41	67.64±19.79	74.74±12.66	0.001**

* Chi square test,

** ANOVA test

**Figure 1:**
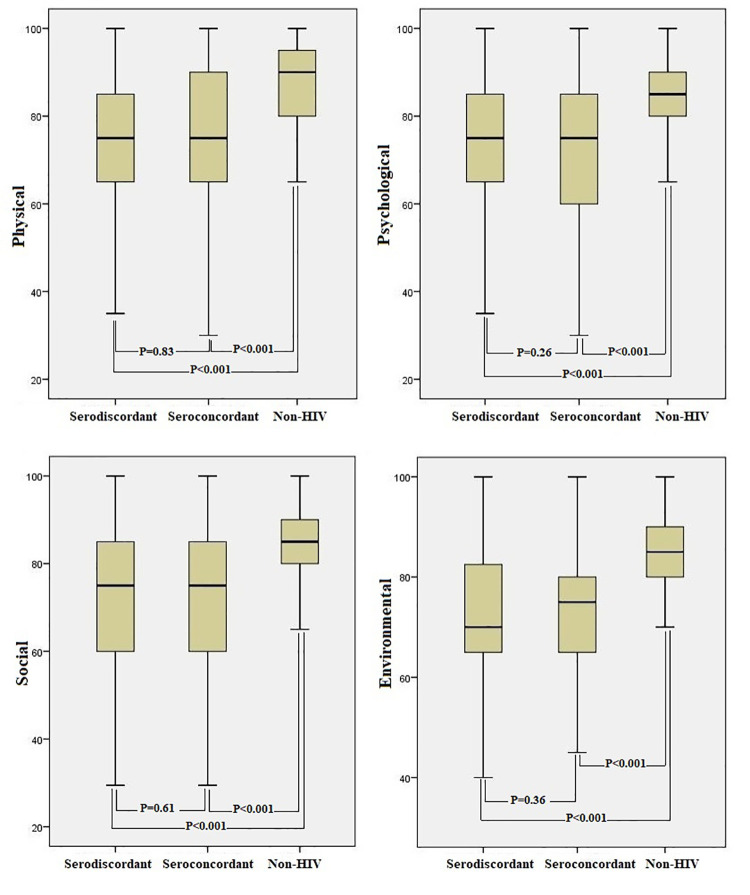
Comparison of the quality of life domains among the study groups. *LSD post hoc test

The score of quality-of-life domains in serodiscordant, seroconcordant and non-HIV couples is displayed in Figure 1. LSD post-hoc test showed that the score of all domains of QOL was significantly higher in non-HIV couples compared to other groups (P<0.001), but no significant difference was observed between total QOL of serodiscordant and seroconcordant groups (P=0.99).

**Figure 2:**
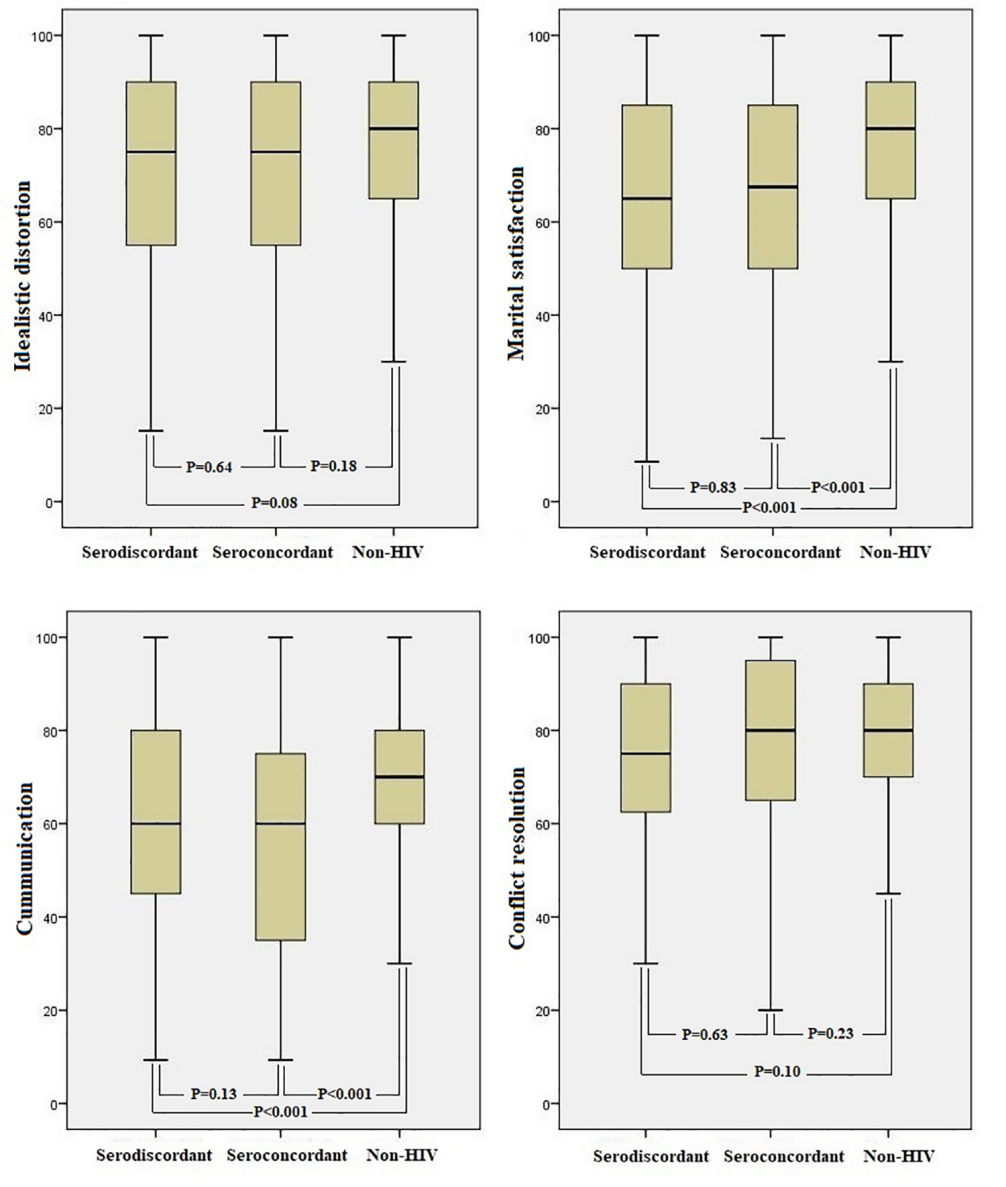
Comparison of marital contentment scales among the study groups. *LSD post hoc test

ANOVA test revealed that there was a significant difference in total marital contentment status among the groups (P=0.001). However, there was no significant difference between the serodiscordantand seroconcordant couples in each area of marital contentment (P=0.79). A significant difference was observed in this aspect between the serodiscordant and seroconcordant in comparison to non-HIV couples (P=0.002 and P=0.001, respectively). The results of each domains are shown in Figure 2.

Table 2 shows the comparison of QOL and marital contentment assessment between HIV positive males and females in all HIV positive patients (serodiscordant and seroconcordant group). As more explanation, the total positive females (96 patients) was the sum of seroconcordant and positive females of serodiscordant groups (74+22). Also, total positive males (118 patients) was calculated by the sum of seroconcordant and positive males of the serodiscordant groups (74+44). The total mean score of QOL in HIV positive females was slightly higher than HIV positive males, but this mentioned difference was not significant (P=0.13). In addition, the total mean score of marital contentment in HIV positive males was significantly higher than HIV positive females (P=0.01). As summarized in Table 3 , the total mean QOL score of HIV positive males of the seroconcordant groups, compared to females, was not significant (P=0.18), but the total mean score of marital contentment in HIV positive males was significantly higher (P=0.01). Besides, in seroconcordant couples, males obtained a significantly higher mean score in idealistic distortion and conflict resolution domains (P=0.03 and P=0.002, respectively). This difference was not seen among males in the serodiscordant group, as shown in  Table 4. It is noticeable that this Table describes gender-based differences just in the serodiscordant couples.

**Table2:** Comparison of the quality of life and marital contentment status between males and females in all HIV positive patients (serodiscordant and seroconcordant groups)

Variable	Group Female (N=96) Male (N=118)	Mean±SD	P value*
Quality of life			
Physical Domain	Female	76.51±16.86	0.15
	Male	73.09±17.20	
Psychological domain	Female	72.81±15.13	0.50
	Male	71.27±18.02	
Social Domain	Female	75.99±15.84	0.17
	Male	72.67±19.63	
Environmental Domain	Female	74.79±12.54	0.06
	Male	71.19±15.13	
Total Quality of Life	Female	75.03±12.40	0.13
	Male	72.06±15.47	
Marital contentment			
Idealistic Satisfaction Scale	Female	68.59±26.98	0.04
	Male	75.51±21.11	
Marital Satisfaction Scale	Female	65.21±24.88	0.06
	Male	70.97±19.83	
Communication Scale	Female	56.09±24.67	0.09
	Male	61.65±23.23	
Conflict Resolution Scale	Female	71.93±24.51	0.01
	Male	80.47±17.39	
Total marital Contentment	Female	65.46±21.34	0.01
	Male	72.15±16.63	

* Independent t-test

**Table3:** Comparison of the quality of life and maritalcontentment status between males and females in the seroconcordant group

Variable	Group Female (N=74) Male (N=74)	Mean±SD	P value*
Quality of life			
Physical Domain	Female	75.88±17.11	0.25
	Male	72.57±17.77	
Psychological domain	Female	71.76±15.14	0.65
	Male	70.47±18.51	
Social Domain	Female	75.88±15.36	0.16
	Male	71.82±19.58	
Environmental Domain	Female	74.80±12.51	0.09
	Male	70.88±15.27	
Total Quality of Life	Female	74.58±12.15	0.18
	Male	71.43±15.75	
Marital contentment			
Idealistic Satisfaction Scale	Female	67.36±28.02	0.03
	Male	76.22±20.92	
Marital Satisfaction Scale	Female	63.31±25.55	0.08
	Male	70.07±20.72	
Communication Scale	Female	53.31±24.98	0.14
	Male	59.12±22.41	
Conflict Resolution Scale	Female	70.27±26.22	0.002
	Male	81.42±16.64	
Total marital Contentment	Female	63.56±21.92	0.01
	Male	71.71±16.57	

* Independent t-test

**Table4:** Comparison of the quality of life and marital status between HIV positive males and females in the serodiscordant group

Variable	Group Female (N=22) Male (N=44)	Mean±SD	P value*
Quality of life			
Physical Domain	Female	78.64±16.20	0.28
	Male	73.98±16.33	
Psychological domain	Female	76.36±14.90	0.39
	Male	72.61±17.31	
Social Domain	Female	76.36±17.74	0.65
	Male	74.09±19.86	
Environmental Domain	Female	74.77±12.95	0.42
	Male	71.70±15.06	
Total Quality of life	Female	76.37±13.67	0.57
	Male	73.10±15.10	
Marital contentment			
Idealistic Satisfaction Scale	Female	72.73±23.23	0.78
	Male	74.32±21.64	
Marital Satisfaction Scale	Female	71.59±21.79	0.86
	Male	72.50±18.38	
Communication Scale	Female	65.45±21.54	0.94
	Male	65.91±24.22	
Conflict Resolution Scale	Female	77.50±16.89	0.77
	Male	78.86±18.67	
Total marital contentment	Female	71.82±18.31	0.81
	Male	72.90±16.90	

* Independent t-test

## DISCUSSION

In the current study, the result showed that QOL and marital contentment were different among non-HIV and HIV infected couples; the status was not different considering the seroconcordant and serodiscordant groups. Besides, marital contentment was higher among males than females of the infected patients. 

Multiple factors can affect HIV such as sociodemo graphic, educational and socioeconomic factors. A study in 2017 indicated that in the serodiscordant relationships men contributed more as the indexed infected person and his spouse was younger in comparison to the other group, while in seroconcordant relationships, both partners were young. ^19^ In our study, the number of infected males was twice that of the infected females in the serodiscordant group, which is in line with them, although our result showed younger couples in the non-HIV group with no statistically significant age difference between the seroconcordant and serodiscordant groups.

Socioeconomic level and educational status are the factors playing a role in HIV infected patient’s contribution to health care units. ^20 , 21^ This fact is similar to our study that non-HIV couples had a higher educational level in comparison to the infected couples. It may be due to more knowledge of the roots of transmission, more prevention and low contact to high-risk individuals in the more educated individuals. Based on this study, prevalence of the disease is affected by their type of marriage. Despite the fact that the most common type of marriage in non-HIV is by family selection with individual’s companionship, in this group, spouses were selected by individual’s options more than other groups. In the seroconcordant and serodiscordant groups, family had a more prominent role in selection of their partner. This can be the indicator of educational discrepancy among the groups. Besides, by checking marriage duration among the groups, non-HIV and seroconcordant couples had passed more married years than the serodiscordant group which can be defined as more desire to live individuals together when they are identical in HIV serum status.

Based on our study, the non-HIV group subjects were born in urban more than rural areas and the affected couples were attributed more to rural areas. It can be explained by the fact that in rural areas, individuals have endogamy marriage with no chance to divorce if they confront with these situations. Furthermore, they may not check their serum status or notice if a person seems to be infected.

A systematic review emphasized on the presence of desire to conceive among the serodiscordant that was attributed fewer living children, younger age, believing partner desire to have child and having no children with the current partner There are some controversies on the effect of using Antiretroviral Therapy (ART) and the sex of indexed partner among serodiscordant relationships on fertility desire. ^22^ We are in line with the authors who mentioned that desire of fertility and having children had not been different among the infected and non-infected groups. Nevertheless, induced abortion is noticed as a trouble among HIV infected couples. ^23^ Phobia of transmission to the child, low economy status, obstetrics complications, and lack of appropriate support can be mentioned as the reasons of this decision among the couples.

QOL is affected by some factors among people living with HIV. Based on our study results which are consistent to another study, QOLis decreased among positive couples in comparison to non-HIV couples in all physical, psychological, social and environmental domains. When assessing QOL among the seroconcordant and serodiscordant groups, we detected no difference. It may be due to the burden of the disease affecting the relationships. Hopelessness is mentioned as one of the influencing factors, especially among the burdens from the lack of mental health units in societies. ^24^ Anxiety and depression are other problems that can create a vicious cycle, especially when alcohol and substance abuse are added to overcome these problems. ^25 , 26^ Fatigue and sleep disturbance can bother the infected patients by disturbing normal performance of the patients. ^27^ Also, cognitive impairment caused by emotional distress can magnify the problem. ^28^ What’s more, immune deficiency secondary to HIV infection leads to increased rate of infections and other morbidities affecting QOL. ^29^ Although technology-mediated interventions can be effective for improvement of QOL, they seem to be insufficient. ^30^ On basis of a survey, eight themes measuring general health, stigma, social support, self-esteem, social support, anxiety, and depression and sleeping disturbance were evaluated by a questionnaire and resulted in the strong relationships between them affecting each other. ^31^

As to the marital contentment, although there was no significant difference in the idealistic distortion and conflict resolution areas in general, infected couples had less contentment than non-infected ones. When evaluating this item among the seroconcordant and serodiscordant groups, we found no difference in this aspect. In other words, both individuals develop marital dissatisfaction although one individual is HIV infected and the other one is serum negative. One explanation may be the effect that one indexed infected individual may have on the relationship. To overcome marital problems among HIV positive patients, we should consider several factors. As to the role of biologic changes of hormonal abnormalities, It seems that sexual hormonal level is in the lower level of normal range in the affected group compared to non-dysfunctional group. ^32^ Fatigue and sleep disturbance are the other factors which reduce marital contentment among affected people. ^27^

Fear of transmission among serodiscordant couples as the major root of transmission should be kept in mind. ^33^ The major wayof HIV transmission in this group is sexual transmission ,blood transfusion, and injection. ^34^ In our study, in line with previous studies, we found that totally sexual root and blood transfusion reconsidered as the major causes of transmission. The evaluation of the subject in subgroups show that, in contrast to results among infected and non-infected patients, it is the major root among seroconcordant relationships, while injection is the major root among the serodiscordant groups. To explain the difference, enhancing the couple’s education, using condom barriers, ^34^ vaginal microbicide, voluntary self-testing and most promisingly early initiation of ART can help the serodiscordant couples not to be infected by sexual transmission. ^35^ To minimize the risk of transmission, we should consider some strategies like consuming ART, unprotected intercourse during peak of female ovulation, intra-uterine insemination, medical male circumcision, and pre-exposure prophylaxis. ^22^

Sex of the infected patient may be an effective variant. Medication adherence, tolerability of drugs and engagement with healthcare providers can be different based on the patient’s sex. ^36 , 37^ Besides, violence against women and increased risk of transmission to seronegative women among serodiscordant couples rather than men reduplicate a woman problem. ^13^ Stigma of infection with HIV in the society and also inappropriate behavior of medical staff can impair the social status of a women more than men. ^38^

When we evaluated QOL and marital contentment in total males and females among infected groups, we found that marital contentment was different among the groups, in contrast to QOL that had no difference. To determine the issue among males and females in the serodiscordant group, no difference was seen in both aspects of QOL and marital contentment. It means that the meaningful difference of marital contentment among total males and females is attributed to the difference among the seroconcordant groups that males have more marital contentment. Lack of fear to transmit the disease to the partner may have a role. What’s more, they may use condom barriers less than the serodiscordant group which may change the sexual enjoyment itself.

A study revealed that sexually dissatisfaction was attributed to orgasm difficulties among infected women, while discouragement, high HIV stigma, being sexually passive in the last 6 months, and a differed unfavorable sex life after HIV diagnosis are mentioned as the major factors of dissatisfaction among the infected men. ^39^ Sexual transmitted disease (STD),being more prevalent among females than males, has a high incidence among the seroconcordant group in comparison to the serodiscordant one. ^34^ Early detection of STD should be kept in mind to decrease the rate of infection in serodiscordant relationships. ^10 , 34^

This study had several strengths including assessing QOL and marital contentment among non-HIV couples and HIV infected couples including seroconcordant, male-infected serodiscordant,and female-infected serodiscordant spouses. The limitation of our study was that the factors affecting QOL of every individual like culture, social status, variation of health system beyond the societies and infection with other sexual transmitted diseases were not included in the study.

## CONCLUSION

Our study revealed that although QOL and marital contentment, especially marital satisfaction scale, are different among non-HIV and HIV infected couples, the status was not different considering the subgroups of seroconcordant and serodiscordant relationships. Besides, marital contentment is considered higher among males rather than females. It magnifies the importance of focusing on woman’s health and psychologic conditions, especially in the serodiscordant groups. It can be achieved by increasing the women and physician’s knowledge, providing more professional healthcare units, more counseling and considering disturbing agents related to HIV infected people. Further studies in other nationalities are necessary to elucidate the effect of limitations of this study by considering other confusing factors.
